# Advances in nursing care for post-stroke limb dysfunction rehabilitation

**DOI:** 10.3389/fneur.2025.1615500

**Published:** 2025-07-24

**Authors:** Jie Bai, Kecheng Chen

**Affiliations:** ^1^Center for Rehabilitation Medicine, Rehabilitation & Sports Medicine Research Institute of Zhejiang Province, Department of Rehabilitation Medicine, Zhejiang Provincial People’s Hospital (Affiliated People’s Hospital), Hangzhou Medical College, Hangzhou, Zhejiang, China; ^2^Center for Rehabilitation Medicine, Department of Neurology, Zhejiang Provincial People’s Hospital (Affiliated People’s Hospital), Hangzhou Medical College, Hangzhou, Zhejiang, China

**Keywords:** stroke, limb dysfunction, rehabilitation motivation, psychology, nursing care

## Abstract

Stroke-induced limb dysfunction has a significant impact on patients’ functional independence and quality of life. While rehabilitation efficacy depends largely on patient motivation, psychological factors often impede treatment adherence and functional recovery. This narrative review synthesizes current evidence on psychological factors affecting rehabilitation motivation, assessment tools, and evidence-based interventions for stroke patients with limb dysfunction. Psychological barriers, including kinesiophobia, diminished self-efficacy, perceived burden, and inadequate social support, significantly predict rehabilitation outcomes. Clinically validated assessment tools demonstrate variable utility across recovery phases, with the Pittsburgh Rehabilitation Participation Scale offering efficiency in acute settings and the Motivation in Stroke Patients for Rehabilitation Scale providing comprehensive evaluation during subacute and chronic phases. Evidence supports multimodal intervention strategies that combine psychological approaches (such as motivational interviewing) with technological innovations (virtual reality, game-based therapy, wearable devices). Clinical implementation should prioritize early psychological screening within 72 h of admission, followed by integrated assessment protocols combining clinician-rated and self-report measures. Furthermore, personalized motivation enhancement protocols tailored to patients’ cognitive status and recovery phase, along with robust interdisciplinary collaboration between rehabilitation nurses, physical therapists, and psychologists, are essential. Ultimately, this integrated approach optimizes rehabilitation outcomes by comprehensively addressing both physical and psychological dimensions of stroke recovery. In the future, studies should emphasize multidimensional analyses that incorporate individual patient characteristics to develop precise rehabilitation interventions, thereby proposing new strategies for optimizing functional recovery in stroke survivors.

## 1 Introduction

Stroke is the second leading cause of death worldwide and the leading cause of death and disability among adults in China (1). It is characterized by a high incidence, disability, mortality, and recurrence (2). In 2019, stroke affected approximately 101 million people worldwide, accounting for 11.6% of all deaths (3). In China, epidemiologic data show that there are approximately 2.5 million new cases of stroke each year, of which more than 1.5 million die, with a disability rate of 75% (4). The pathogenesis of stroke is complex and can be clinically divided into ischemic and hemorrhagic subtypes (5). Both types can lead to severe sequelae, including physical, sensory, cognitive, and swallowing deficits, and disease progression may exacerbate cardiovascular complications, thereby increasing the risk of death (6). Although advances in medical technology have reduced stroke mortality, this progress has paradoxically led to an increase in disability.

The World Health Organization (WHO) has emphasized the importance of stroke rehabilitation in improving survival rates and the quality of life of stroke patients (7). Rehabilitation is a crucial component in post-stroke recovery, focusing not only on restoring motor functions but also addressing psychological factors, including the prevention of depression, anxiety, and fear of movement, which can hinder rehabilitation efforts. Rehabilitation programs are often guided by national and international stroke rehabilitation nursing guidelines, such as those published by the WHO and the Chinese Medical Association Stroke Branch (8). These guidelines emphasize the importance of holistic and multidisciplinary approaches to stroke rehabilitation, encompassing physical therapy, psychological support, and social reintegration. Despite the recognition of rehabilitation’s importance, a significant gap remains between rehabilitation practices and the optimal outcomes that could be achieved for stroke survivors.

Limb dysfunction occurs in 70–80% of stroke survivors, manifesting as unilateral limb weakness, clumsiness, numbness, limited mobility, and facial asymmetry, which seriously affects the quality of life and activities of daily living, and at the same time brings a heavy burden to the family (9, 10). Systematic and standardized rehabilitation care is the cornerstone of promoting patients’ functional recovery, effectively enhancing muscle strength, balance, and motor ability, and ultimately accelerating the recovery process and improving the quality of life (11). Studies have shown that such patients often experience significant negative psychological states during the rehabilitation process, especially the fear of re-injury and pain, which seriously affects the treatment outcome (12).

Central to the effectiveness of rehabilitation is the concept of rehabilitation motivation, defined as the psychological drive that encourages stroke patients to engage actively in their rehabilitation program (13). Research has shown that rehabilitation motivation is a key determinant of treatment adherence and functional recovery. Motivation is closely linked to a variety of factors, including sociodemographic characteristics (such as age, education, and marital status), disease-related factors (such as the location of the stroke lesion and disease duration), psychosocial factors (such as family support and psychological well-being), and self-efficacy (14). Furthermore, emerging technologies such as virtual reality (VR) and robotic rehabilitation devices have shown promise in increasing patient engagement and motivation, leading to improved rehabilitation outcomes (15, 16). To better understand the mechanisms behind rehabilitation motivation, psychological models such as the Self-Determination Theory (SDT) offer valuable insights. According to SDT, motivation is influenced by the fulfillment of three basic psychological needs: autonomy, competence, and relatedness (17). Autonomy refers to the feeling of control over one’s actions, competence involves the sense of mastery over tasks, and relatedness encompasses the connection with others, such as family and healthcare providers (18). These intrinsic motivators can significantly enhance a stroke patient’s willingness to engage in rehabilitation activities, leading to improved adherence and recovery. Understanding these psychological principles is crucial for designing rehabilitation programs that not only address the physical limitations of stroke survivors but also foster intrinsic motivation for recovery.

In addition to psychological factors, rehabilitation motivation is also influenced by the design and implementation of rehabilitation programs. Structured rehabilitation programs that incorporate tailored interventions, personalized goal setting, and patient-centered care approaches have been shown to enhance motivation (19). The role of family and social support is also critical, as strong social connections have been shown to improve motivation and compliance with rehabilitation regimens (20). Moreover, the integration of new rehabilitation technologies, such as tele-rehabilitation and VR-based therapies, enables more engaging, accessible, and personalized care, which can further enhance motivation for rehabilitation.

Given the multidimensional nature of rehabilitation motivation, it is essential to assess both physical and psychological factors comprehensively. This review aims to explore the factors influencing rehabilitation motivation in stroke patients, evaluate emerging assessment tools for both physical dysfunction and psychological well-being, and propose innovative nursing interventions that can optimize rehabilitation outcomes. The goal is to provide evidence-based strategies that healthcare providers can use to enhance motivation, improve rehabilitation adherence, and ultimately promote better functional recovery for stroke survivors.

## 2 Psychological determinants of rehabilitation in patients with post-stroke limb dysfunction

Adverse psychological states associated with falls in patients with stroke physical dysfunction are essential factors affecting functional recovery and increasing fall risk, with fear being one of the most common psychological manifestations. Fear is influenced by age, education, previous history of falls, rehabilitation nursing education, rehabilitation exercise self-efficacy, self-perceived burden, social support, rehabilitation exercise self-efficacy, and co-morbid anxiety and depression (21, 22).

### 2.1 Sociodemographic factors

Age, education, income, and marital status were the main sociodemographic factors influencing the motivation of stroke patients to rehabilitate (23, 24). Kim et al. identified age and education as predictors of motivation to rehabilitate (25). Younger and middle-aged patients had stronger motivation for rehabilitation compared to older patients, possibly due to their greater family and social responsibilities, which prompted greater adherence to exercise to accelerate return to family and community roles. Meanwhile, studies have shown elevated levels of agoraphobia in the elderly population (26). It is essential to clarify the term “agoraphobia” in the context of stroke rehabilitation. While “agoraphobia” is traditionally defined in the clinical psychiatric sense as a fear of open spaces or situations where escape might be intricate, in stroke rehabilitation literature, it often refers to fear of movement or exposure to physical activity (27). This fear, also known as kinesiophobia, can manifest as avoidance behaviors, where stroke survivors resist engaging in physical rehabilitation exercises due to the fear of re-injury or pain. As such, the rehabilitation process must carefully address both the psychological components of agoraphobia and the fear of movement, especially in older stroke patients, through targeted interventions and education to mitigate these fears.

In recent research, Lee et al. revealed that age was an independent predictor of fear of falling at all stages of stroke, with a 1.6-fold increase in the prevalence of fear of falling for every 10-year increase in age above 60 years (28). Advanced age in stroke patients with physical dysfunction was associated with decreased limb coordination, visual and auditory dysfunction, and increased fear of rehabilitation-related adverse events. Furthermore, a systematic review concluded that the elderly population’s fear of falling is a significant predictor of poor rehabilitation adherence and negative psychological outcomes such as depression and anxiety (29).

Educational attainment is a fundamental demographic parameter that can enhance confidence in rehabilitation by improving health literacy (30). Patients with higher levels of education have a more comprehensive knowledge base and are more likely to understand the etiology of the disease and the necessity of rehabilitation. Researchers identified per capita monthly household income as a key predictor of motivation to rehabilitate, which may be mediated by increased access to rehabilitation resources and reduced financial stress in higher-income households (31). Marital status further moderated rehabilitation motivation, with married patients in harmonious marital relationships having stronger motivation for rehabilitation and lower fear of falling than unmarried or caregiverless individuals (32). Spousal support reduces psychological distress (e.g., pain perception, anxiety) and strengthens commitment to rehabilitation through emotional encouragement (33).

Therefore, clinicians should design individualized rehabilitation programs based on these sociodemographic variables (age, income, education, and marital status) to meet the needs of different patients and optimize motivation for rehabilitation.

### 2.2 Clinical factors

Disease-related factors such as disease duration and lesion site significantly influenced the level of motivation for rehabilitation in stroke patients (34). Kobylanska et al. demonstrated that prolonged disease duration was associated with decreased motivation for rehabilitation, possibly due to a diminished belief in the efficacy of rehabilitation, as patients perceived limited improvement in functioning compared to previous interventions (35). In contrast, Tan et al. reported higher motivation for rehabilitation in patients with bilateral lesions compared to those with unilateral lesions (36). This may reflect the need for intensive bilateral case neurorehabilitation and motor skill reacquisition, potentially activating patients’ intrinsic motivation and self-efficacy. Yoshida et al. found that multisystem symptoms (e.g., fatigue, weakness, pain) were negatively correlated with adherence to rehabilitation through reduced participation in therapeutic exercise in a qualitative study of 65 subacute stroke patients (37, 38).

Fall history and caregiver dynamics further moderated psychological status in patients with stroke physical dysfunction (29, 39). Evidence suggests that prior fall events exacerbate kinesiophobia and amplify fears during rehabilitation. Falls reinforce patients’ perceived risk of motion-induced injury and adverse outcomes (e.g., secondary injury, prolonged recovery time), which increases the financial burden and caregiver stress (40). As a result, those who experience falls tend to exhibit avoidance behaviors or resist rehab exercises, leading to poor compliance and delayed functional recovery, thus creating a vicious cycle.

Cognitive impairment and post-stroke fatigue also present significant barriers to rehabilitation participation. Cognitive impairment, commonly observed after stroke, affects memory, attention, and executive functions, all of which are essential for participating in and adhering to rehabilitation programs. A recent cohort study by Kobylanska found that cognitive decline was significantly associated with lower motivation to engage in rehabilitation, as patients with cognitive deficits struggle with tasks that require sustained attention and problem-solving (35). Moreover, post-stroke fatigue, often experienced as overwhelming tiredness not alleviated by rest, is a common complaint among stroke survivors. This fatigue can severely limit a patient’s ability to participate in physical therapy, as it diminishes energy levels and increases perceived effort during exercises. In a longitudinal observational cohort study, nearly 40% of stroke survivors reported that fatigue reduced their motivation for rehabilitation, highlighting the importance of addressing fatigue as part of a comprehensive rehabilitation program (41).

In clinical practice, healthcare professionals should prioritize patients with prolonged disease duration, bilateral lesions, or multisystem symptoms (42). Timely education of patients about disease mechanisms and rehabilitation strategies, combined with lesion/symptom-specific interventions, is essential to reduce symptom burden and increase confidence in recovery. Clinicians should also consider integrating cognitive training and fatigue management techniques alongside traditional rehabilitation exercises.

### 2.3 Rehabilitation nursing and health education interventions

Higher frequency of exercise and prolonged participation were associated with increased motivation, leading to more active involvement and self-efficacy during rehabilitation (43). Evidence suggests that the professional competence and patient-centeredness of the rehabilitation care team play a critical role in the development of motivation for rehabilitation in stroke patients (44). This may be attributed to evidence-based rehabilitation protocols, individualized care strategies, and ongoing psychosocial support provided by high-performing teams, all of which increase patient adherence and optimism (45). A systematic review further showed that advanced rehabilitation technologies, such as virtual reality (VR) technology and tele-rehabilitation systems, significantly improved motivation (46). This effect may be attributed to the fact that these technologies improve accessibility to treatment, reduce kinesiophobia (fear of movement), and create an engaging, goal-oriented rehabilitation experience that promotes continued patient engagement.

Constructing a rehabilitation education program is essential for patients and their families to provide knowledge about rehabilitation, self-management skills, and caregiver training (47, 48). Inadequate health education affects health literacy, leading to limited awareness of the functional benefits of rehabilitative exercise, decreased treatment adherence, increased agoraphobia, and poorer outcomes the more severe the fear of exercise. Clinically, healthcare professionals should implement an individualized goal-setting framework to systematically improve the frequency and duration of exercise, thereby enhancing motivation to exercise. In addition, integrating advanced rehabilitation equipment (e.g., robotic assistive devices) and establishing a peer support network can enhance patients’ confidence, reduce psychological barriers, and ultimately improve functional recovery and quality of life.

### 2.4 Psychosocial factors

Psychosocial factors (including personality traits, negative emotional states, family support, and social support) significantly moderated psychological status and functional recovery in stroke patients (49). Exacerbation of agoraphobia (fear of movement) was associated with reduced rehabilitation motivation, possibly due to fear-induced treatment resistance, reduced confidence in recovery, and subsequent poor treatment outcomes. Notably, extroverted personality traits were positive predictors of motivation, which may be attributed to enhanced stress management and more effective coping strategies with illness in these individuals. A study found that stroke patients with higher extraversion scores exhibited greater adherence to rehabilitation programs and better outcomes compared to introverted individuals. This could be attributed to the extrovert’s tendency to seek social support, remain optimistic, and engage in positive interactions with healthcare providers and family members (50). Empirical evidence confirms that strong family support enhances rehabilitation motivation in stroke survivors (51).

Social support, defined as multidimensional help (e.g., financial, emotional, informational), is an essential protective factor for rehabilitation motivation after stroke (52). It promotes positive health behaviors, attenuates agoraphobia, and maintains optimism about clinical prognosis. Lee et al. demonstrated a positive correlation between navigating social support and rehabilitation motivation, with higher levels of support enabling patients to access disease-specific resources and emotional recognition, leading to increased engagement (53). The 1 ~ 3 months after stroke is a critical period for treating fall-related anxiety; stronger social support improves patients’ emotional well-being, resilience, and treatment adherence. Clinician-directed interventions during this phase can enhance exercise confidence, mitigate negative emotions, and expedite functional recovery.

Therefore, healthcare providers should prioritize psychosocial interventions that target family dynamics and community support systems. The incorporation of personality assessments into rehabilitation care planning can help tailor interventions that align with the patient’s psychological profile. Integrating multidisciplinary strategies such as cognitive-behavioral approaches, family-centered education, and social resource mobilization is essential to address emotional distress, optimize motivation for rehabilitation, and improve long-term quality of life in stroke survivors.

### 2.5 Exercise self-efficacy and self-perceived burden

Exercise self-efficacy and self-perceived burden are essential influences on the rehabilitation of stroke patients with physical dysfunction (54). Enhanced motor self-efficacy improves treatment adherence, promotes active participation in rehabilitation programs, and accelerates functional recovery (55). In contrast, low exercise self-efficacy reflects the psychological impact of motor deficits and is manifested by exercise avoidance behaviors, decreased confidence in completing rehabilitation tasks, and increased agoraphobia. Disease perceptions and coping strategies moderated exercise self-efficacy in stroke patients, whereas rehabilitation exercise self-efficacy was negatively associated with fear. Higher rehabilitation exercise self-efficacy was associated with lower levels of anxiety, whereas lower self-efficacy was associated with therapeutic pessimism, reduced disease resilience, and increased fall-related fear during rehabilitation activities (56). Patients with elevated self-efficacy demonstrated greater resilience, proactive rehabilitation participation, and better functional outcomes. Strategies such as peer modeling and cognitive restructuring have been proven effective in enhancing self-efficacy. Peer modeling, where patients observe others who have successfully overcome similar rehabilitation challenges, provides social proof and motivation. Cognitive restructuring, on the other hand, involves challenging negative thoughts and replacing them with positive, realistic beliefs about one’s abilities, which can reduce anxiety and foster a more proactive approach to rehabilitation.

Moderate to high levels of self-perceived burden can exacerbate agoraphobia, especially when motor deficits are exacerbated, delaying the recovery process, impairing activities of daily living, and increasing the need for family care. To reduce perceived risk, patients often resort to motor avoidance or refusal, which may escalate into pathological agoraphobia. Oh et al. found that depression and anxiety co-morbidity negatively impacted motivation for rehabilitation (57). Anxiety was positively correlated with fear and was a key predictor of fall-related fear in mobility tasks. Severe anxiety was associated with mood dysregulation, maladaptive coping strategies, and longer rehabilitation times, thereby prolonging fall risk.

Clinical interventions should include peer mentoring programs, in which patients who have successfully recovered share their narratives of their experiences to enhance self-efficacy and reduce anxiety. Multimodal educational tools (e.g., animated presentations, therapeutic music) may improve understanding of motor rehabilitation programs, thereby increasing treatment adherence. For agoraphobia, rehabilitation should implement graded task-oriented training, starting with basic activities of daily living skills (e.g., independent eating, grooming) and progressing to full functional recovery.

## 3 Rehabilitation motivation assessment tools for stroke patients

Rehabilitation motivation is a significant predictor of treatment outcome in stroke patients. Practical assessment tools not only quantify patients’ motivation for rehabilitation and increase their confidence, but also improve treatment adherence by setting milestones. When combined with psychological interventions, these tools can optimize quality of life, promote motor recovery, and reduce the risk of long-term disability, ultimately achieving a dual goal: optimizing healthcare resource allocation and maximizing individual rehabilitation benefits (Table 1).

**Table 1:** Rehabilitation motivation assessment tools for stroke patients.

Assessment tool	Purpose	Domains/items	Scoring method	Strengths	Limitations
Intrinsic Motivation Inventory (IMI)	Assess intrinsic motivation	7 domains (interest, perceived competence, etc.); 45 items	7-point Likert scale (1–7)	Multidimensional, sensitive to interventions	Time-intensive, disputed subscale validity, limited validation in stroke
Pittsburgh Rehabilitation Participation Scale (PRPS)	Assess rehabilitation participation in hospitalized patients	6 levels (refusal to excellent participation)	Clinician-rated observation	Rapid; cross-language applicability (ICC > 0.75)	Lacks patient self-report; overlooks physiological factors
Motivation for TBI Rehabilitation Questionnaire (MOT-Q)	Assess rehabilitation motivation in TBI patients	4 domains (denial, interest, anger, professional reliance); 31 items	5-point Likert scale (−2 to +2); total score: −62 to +62	Guides personalized interventions	Overlooks intrinsic motivation; response bias in cognitively impaired patients
Stroke Rehabilitation Motivation Scale (SRMS)	Stroke-specific rehabilitation motivation assessment	7 domains; 28 items	5-point Likert scale (1–5); reverse scoring for items 5, 12, 23	Disease-specific design; high reliability (Cronbach’s α > 0.75)	Static assessment; bias in aphasia/cognitive impairment
Motivation in Stroke Patients for Rehabilitation Scale (MORE)	Assess goal-oriented rehabilitation motivation	3 domains (interpersonal, social, behavioral); 17 items	7-point Likert scale (1–7); total score: 17–119	High reliability (Cronbach’s α = 0.983); validated Chinese version	Limited to first-episode stroke; lacks data in severe impairment

### 3.1 Intrinsic motivation inventory

Initially developed by Ryan et al. in 1983 and subsequently validated by McAuley et al. (58), the IMI is the most widely used instrument for assessing levels of intrinsic motivation in clinical populations. This 45-item instrument assesses seven dimensions: interest/enjoyment, perceived competence, effort, value/usefulness, stress/strain, relevance, and perceived choice. Each item is rated on a 7-point Likert scale (1 = Strongly Disagree to 7 = Strongly Agree), with higher total scores indicating higher levels of intrinsic motivation. Key strengths include a multidimensional structure, robust reliability and validity (Cronbach’s *α* > 0.8), broad applicability (e.g., education, rehabilitation), and sensitivity to intervention effects. Limitations include susceptibility to the social desirability bias inherent in self-report measures, time-intensive administration due to the large number of items, and the controversial validity of some subscales (59). The applicability of the IMI in stroke populations requires further validation.

### 3.2 Pittsburgh Rehabilitation Participation Scale

The PRPS, developed by Lenze et al. (60), is a clinician-assessment tool designed to evaluate motivation levels during rehabilitation by observing the frequency of inpatient participation in treatment and attitudes toward treatment. Rehabilitation participation is categorized into six levels of refusal, poor, fair, good, very good, and excellent, with higher scores indicating greater motivation for rehabilitation (61). The PRPS has been adapted into several languages (e.g., Italian, Swedish) and is widely used in clinical settings. Its strengths include a simple structure, rapid administration, and robust psychometric properties (ICC > 0.75), making it suitable for stroke, spinal cord injury, and other rehabilitation contexts. However, as a purely observational tool, the PRPS lacks a patient self-report component and may ignore the impact of individual physiologic conditions and disease progression on motivation, potentially compromising its ability to reflect, authentic patient engagement.

The IMI provides deeper insight into motivational constructs, but requires intact cognitive and linguistic abilities, whereas the PRPS offers efficient clinical utility with less dimensional depth. Clinicians should select between these complementary tools based on assessment objectives, patient characteristics, and available resources: IMI for comprehensive assessment when cognitive function permits or in research settings, and PRPS for efficient routine clinical monitoring, especially in patients with communication challenges (Table 2).

**Table 2:** Intrinsic motivation inventory (IMI) vs. Pittsburgh Rehabilitation Participation Scale (PRPS): comparative analysis.

Feature	Intrinsic motivation inventory (IMI)	Pittsburgh rehabilitation participation scale (PRPS)
Administration	Self-report	Clinician-rated
Assessment focus	Multidimensional intrinsic motivation (interest/enjoyment, perceived competence, effort, value/usefulness)	Observable participation behavior and attitude during therapy sessions
Structure	45 items across seven dimensions	6-point single-item scale
Time required	15–20 min	1–2 min
Strengths	Comprehensive assessment of motivational constructs; captures subjective experience	Rapid administration; minimal patient burden; captures observable behavior
Limitations	Susceptible to social desirability bias; cognitive demands may limit use in patients with aphasia	Limited assessment of motivational dimensions; potential observer bias
Optimal application	Comprehensive assessment when cognitive function permits; research settings	Routine clinical monitoring; patients with communication difficulties

### 3.3 Motivation for traumatic brain injury rehabilitation questionnaire

The MOT-Q was developed by Chervinsky et al. (62) to assess motivation for rehabilitation in patients with traumatic brain injury (TBI) after the acute phase. The scale consists of 31 entries in 4 dimensions: non-denial, interest in rehabilitation, lack of anger, and dependence on professional help. Each item is scored on a 5-point Likert scale (−2 = Strongly Disagree, + 2 = Strongly Agree; 0 = Not Applicable), with a total score ranging from −62 to ~ + 62, with higher scores being associated with stronger motivation for rehabilitation. Key strengths include the targeted assessment of motivation-related constructs (e.g., goal setting, self-efficacy), multidimensional constructs, satisfactory reliability (Cronbach’s *α* > 0.75), and utility in guiding individualized interventions (63). Limitations include reliance on self-report (patients with cognitive impairment or illness sense dissonance are susceptible to response bias) and a focus on extrinsic motivational factors at the expense of intrinsic motivational mechanisms. The MOT-Q is primarily suited for motivational screening and intervention optimization in patients with moderate to severe TBI.

### 3.4 Stroke Rehabilitation Motivation Scale

The SRMS was developed from the Sports Motivation Scale (SMS) in 2012 to assess motivation for rehabilitation in stroke patients. The scale consists of 28 items assessing 7 dimensions on a 5-point Likert scale (1–5), with higher total scores being associated with greater motivation for rehabilitation. Scores for items 5, 12, and 23 are reversed (64). Its main strengths include its disease-specific design, concise structure, and robust psychometric properties (Cronbach’s *α* > 0.75), which enable the identification of individuals with problematic motivation and guide targeted interventions. It remains one of the most practical tools for assessing motivation in stroke rehabilitation. However, in patients with cognitive impairment or aphasia, reliance on self-report introduces a potential response bias. In addition, its static assessment focus cannotto track fluctuations in motivation during rehabilitation dynamically. It is recommended that the SRMS be integrated with clinical observations or objective behavioral indicators (e.g., adherence logs) to improve accuracy and make it suitable for clinical motivation screening and intervention optimization.

### 3.5 Motivation in Stroke Patients for Rehabilitation Scale

The MORE scale was developed by Japanese researcher Yoshida, based on a pool of entries created by nine rehabilitation specialists, to assess motivation for rehabilitation in stroke patients (64). The scale consists of 17 entries that include three dimensions: interpersonal, social, and behavioral change. Each entry is scored on a 7-point Likert scale (1 = strongly disagree to 7 = strongly agree) with a total score range of 17 ~ 119. Higher scores indicate greater rehabilitation motivation. Strengths include a focus on recovery confidence and goal orientation, a concise structure, and robust psychometric properties (Cronbach’s *α* > 0.75), as well as the ability to identify motivational deficits and provide guidance for interventions. In 2023, Tan et al. validated the Chinese version of the MORE, with a Cronbach’s α coefficient of 0.983 and a retest reliability (ICC) of 0.994 (65). Limitations include limited generalizability of the study to the first stroke population, unclear validity in recurrent stroke cases, and the inclusion of mainly patients with high baseline activities of daily living (ADL) ability. Meanwhile, several psychometric limitations warrant consideration. First, its validation has primarily focused on first-time stroke patients with relatively high baseline ADL abilities, limiting generalizability to recurrent stroke cases or severely impaired populations. Second, while the scale demonstrates excellent internal consistency (Cronbach’s *α* > 0.75) and test–retest reliability, its construct validity requires further examination, particularly regarding its ability to differentiate between intrinsic motivation (an internal drive for rehabilitation) and extrinsic motivation (external rewards or pressure). To improve the comprehensiveness of the assessment, further validation and integration of behavioral metrics in severely impaired stroke cohorts are needed. MORE is currently applicable to clinical screening and the development of individualized rehabilitation strategies.

It is worth noting that assessment tools for rehabilitation motivation vary in their validation status for stroke populations. The SRMS and MORE were specifically developed and validated for stroke populations. In contrast, the IMI originated from sports psychology, whereas the MOT-Q was adapted from contexts related to TBI rehabilitation. The PRPS was initially developed for general rehabilitation settings and subsequently validated across various neurological conditions, including stroke.

## 4 Assessment tools for stroke patients with limb impairment

Standardized assessment tools for post-stroke limb dysfunction, such as the Fugl-Meyer Motor Function Assessment (FMA) and the Brunnstrom Motor Function Recovery Staging (9, 66), provide the opportunity for precise functional localization and stratification of recovery periods in clinical practice (Table 3). These instruments provide evidence-based guidance for the development of individualized rehabilitation programs (e.g., antispasticity therapy, optimal balance training), as well as facilitating longitudinal monitoring of treatment outcomes for modification of treatment regimens.

**Table 3:** Assessment tools for stroke patients with limb impairment.

Assessment tool	Purpose	Domains/items	Scoring method	Strengths	Limitations
Fugl-Meyer Assessment (FMA)	Evaluate post-stroke motor function	Motor, sensory, balance domains	Performance-based scoring; total score: 0–100	High reliability/validity (ICC > 0.8); sensitivity; guides rehabilitation	Time-intensive (45–60 min); weak ADL correlation; requires specialized training
Tampa Scale for Kinesiophobia (TSK)	Assess kinesiophobia (pain-related fear)	Unidimensional; 17 items	4-point Likert scale (1–4); total score: 17–68; ≥38 indicates clinical significance	Concise structure, rapid screening of exercise avoidance	cultural adaptation challenges; reliance on self-report; lacks behavioral/physiological metrics
Motor Self-Efficacy Scale (MSES)	Assess motor self-efficacy	3 domains (task completion, problem-solving, time management); 9 items	11-point Likert scale (0–10); total score: 0–90	Cross-population applicability; high reliability (Cronbach’s α > 0.8)	Self-report bias; lacks granularity in exercise intensity/frequency
Self-Perceived Burden Scale (SPBS)	Assess psychological burden from care dependency	3 domains (physical, emotional, economic); 10 items	5-point Likert scale (1–5); total score: 10–50; >20 indicates high burden	Sensitive to emotional states in chronic/terminally ill patients	Overlooks objective economic burden; requires complementary tools
Social Support Rating Scale (SSRS)	Assess social support levels	3 domains (subjective, objective; support utilization); 10 items	Total score: 11–72; ≥24 indicates high support	Multidimensional reflection of social networks	Subjective recall bias; high scores may mask qualitative disparities
Morse Fall Scale (MFS)	Assess fall risk in hospitalized patients	6 items; total score: 0–125	Risk tiers: <25 (no risk); 25–45 (low risk); >45 (high risk)	Rapid administration (120–180 s); high predictive validity (AUC > 0.7)	Ignores pharmacological/environmental factors; subjective gait assessment
Self-Efficacy for Rehabilitation Exercise Scale (SER)	Assess rehabilitation exercise self-efficacy	2 domains (physical exercise, coping efficacy); 12 items	0–10 per item; total score: 0–120	Guides interventions, applicable to postoperative/chronic conditions	Social desirability bias; lacks pain tolerance assessment
Hospital Anxiety and Depression Scale (HADS)	Screen anxiety/depression	2 subscales (anxiety, depression); 7 items each	0–3 per item; total score: 0–21 per subscale; ≥9 indicates clinical significance	Excludes somatic symptoms; high reliability (Cronbach’s α > 0.8)	May miss somatic depression; subjective reporting bias
Stroke-Specific Quality of Life Scale (SS-QOL)	Evaluate HRQoL in stroke survivors	12 domains (physical strength, language, etc.); 49 items	5-point Likert scale; higher scores indicate better HRQoL	Stroke-specific; multidimensional	Time-intensive; excludes financial burden

### 4.1 Fugl-Meyer Assessment

The Fugl-Meyer Assessment of Motor Function (FMA) is one of the most widely used quantitative tools for evaluating motor function after stroke (67). Patients with higher stroke severity and poorer limb motor performance had more severe agoraphobia. Lower FMA scores were associated with more severe motor deficits, a reduced ability to exercise in early rehabilitation, increased dependence on external assistance, and an increased risk of falls, which collectively led to reduced rehabilitation participation, reduced self-efficacy, and marked exercise avoidance behaviors (68).

Key strengths include multidimensional assessment (motor, sensory, and balance domains), high reliability and validity (ICC > 0.8), sensitivity in detecting mild to moderate functional changes, and clinical utility in guiding rehabilitation programs. Limitations include a long administration time (~45 to 60 min), a weak correlation with real-world activities of daily living (ADLs), and the need for specialized training to ensure accuracy. Although indispensable in clinical and research settings, the FMA is often combined with other scales to fill gaps in functional activity assessment.

### 4.2 Tampa Scale for Kinesiophobia

In clinical practice, the Tampa Scale for Kinesiophobia (TSK) has been widely adopted as a valid tool for assessing pain-related fear beliefs (69, 70). The scale has a simple structure, high reliability (Cronbachto screen’s *α* > 0.7 quickly), and is capable of rapidly screening for motor avoidance behaviors, making it suitable for use in populations recovering from chronic pain or post-surgery. However, its limitations include challenges of cross-cultural adaptation (ambiguous wording of entries in specific cultural contexts), over-reliance on subjective reports, and lack of integration of behavioral observations or physiological parameters. A comprehensive assessment requires combining the TSK with clinical interviews or supplemental assessment tools.

### 4.3 Motor Self-Efficacy Scale

The MSES consists of 3 dimensions: task completion effectiveness, problem-solving effectiveness, and time management effectiveness (66). The total score (0–10 for each entry) was calculated as the mean score of the entries, with higher scores indicating greater self-efficacy. Key strengths include its concise structure, high reliability and validity (Cronbach’s alpha > 0.8), and applicability across diverse populations (e.g., chronic disease cohorts). Limitations include susceptibility to subjective bias and the social desirability effect inherent in self-reported metrics, as well as insufficient subtle differentiation between exercise modalities or intensity levels in some iterations. The integration of behavioral logs or objective activity monitoring (e.g., accelerometry) is recommended to enhance the accuracy of the assessment.

### 4.4 Self-Perceived Burden Scale

The Self-Perceived Burden Scale (SPBS) was designed to assess psychological distress associated with caregiving dependence (71, 72). The Chinese version of the SPBS scale was used to determine the three dimensions of physical, emotional, and financial burden on a Likert scale (never (1) to always (5)), with a total of 10 entries. The total score ranges from 10 to 50, with a score of ≤20 indicating no or low burden and a score of>20 indicating moderate to high burden (73). Key strengths of the scale include its concise structure, robust reliability and validity (Cronbach’s *α* > 0.75), and sensitivity in detecting emotional states in patients with chronic or terminal illnesses. Limitations include reliance on subjective reporting (susceptibility to social desirability bias, e.g., underreporting of true feelings), overemphasis on the affective domain rather than objective quantification of financial/caregiving burdens, and the need for integration of complementary instruments (e.g., quality of life indicators, caregiver assessments) for a comprehensive patient-caregiver dichotomous analysis.

### 4.5 Social Support Rating Scale

The Social Support Rating Scale assesses patients on three dimensions: subjective support, objective support, and support utilization (74, 75). Strengths include a concise structure, good reliability (Cronbach’s *α* > 0.7), and multidimensionality that reflects social network characteristics. Limitations include reliance on subjective recall, incomplete coverage of support domains, high scores may mask differences in qualitative support, and the need to use supplemental instruments (e.g., qualitative interviews) to characterize the actual utility and emotional value of support.

### 4.6 Morse Fall Scale

The Morse Falls Scale (MFS) is a rapid tool for assessing fall risk on admission to hospital (76). Its clean design and simplicity allow it to be completed in 120–180 s, minimizing the number of personnel required for valid risk stratification, especially in the elderly population. The scale has good reliability and predictive validity (AUC > 0.7), and the low/high risk jointly indicating susceptibility to falls. Limitations include inadequate consideration of pharmacologic and environmental risk factors, reliance on subjective clinical judgment for gait assessment, the controversial validity of entry weights, and saturation of scores among older adults with comorbidities.

### 4.7 Self-Efficacy for Rehabilitation Exercise Scale

The Self-Efficacy for Rehabilitation Exercise Scale (SER) assesses patients’ confidence in completing therapeutic exercise across two dimensions: physical exercise efficacy and coping efficacy (77). Strengths include a concise structure (typically ≤ 12 entries), good reliability (Cronbach’s *α* > 0.75), broad applicability (e.g., postoperative or chronic illness rehabilitation), and practical guidance for targeted intervention strategies. Limitations include susceptibility to social desirability bias (e.g., overestimation of ability) inherent in self-reported metrics, as well as an insufficiently detailed assessment of exercise intensity, frequency, or pain tolerance. The integration of objective exercise logs or behavioral observations is recommended to improve the reliability of assessments.

### 4.8 Hospital Anxiety and Depression Scale

The HADS is primarily used to screen for mood disorders and consists of two subscales: anxiety and depression (78). A threshold score of ≥9 indicates a clinically meaningful level of anxiety or depression, with higher scores reflecting more severe symptoms. Its main strengths are the exclusion of somatic symptoms, its concise structure, high reliability (Cronbach’s *α* > 0.8), and its broad applicability (e.g., in chronically ill or postoperative populations). Limitations include reliance on subjective reports (susceptibility to cognitive bias and social desirability effects), a primary focus on affective symptoms (which may miss depression with prominent somatic manifestations), and the need to use complementary diagnostic methods such as clinical interviews or supplementary scales. Dynamic monitoring of symptom trajectories is recommended to improve diagnostic accuracy.

### 4.9 Stroke-Specific Quality of Life Scale

The SS-QOL was developed by Williams et al. in 1999 as a self-assessment tool to evaluate health-related quality of life (HRQoL) in stroke survivors (79, 80). Each item is scored on a 5-point Likert scale, with higher total scores indicating better overall HRQoL. Key strengths include multidimensional assessment, high reliability and validity (Cronbach’s *α* > 0.8), and disease-specific attention to stroke-related impairments (e.g., speech deficits, upper limb dysfunction). Limitations included reliance on subjective reports (which are prone to response bias in patients with cognitive impairment), time-intensive dosing due to the number of items, and exclusion of external determinants such as financial burden. Supplemental caregiver interviews and longitudinal rehabilitation monitoring are recommended to improve the accuracy of assessments.

### 4.10 Limitations of assessment tools in post-stroke population

Assessment tools face significant challenges when applied to stroke populations. Cognitive impairments, including attention deficits, memory dysfunction, and executive function disorders, may compromise comprehension of complex instructions and accurate self-reporting. Aphasia, affecting approximately 30% of stroke survivors, presents particular challenges for self-report measures, potentially leading to response bias or incomplete assessments. Additionally, anosognosia (impaired awareness of deficits) affects 10–17% of stroke patients, resulting in unreliable self-assessment. Fatigue and fluctuating symptoms further complicate consistent reporting. Cultural and educational factors may influence the interpretation of assessment items, particularly in scales with limited cross-cultural validation.

To address these limitations, clinicians should: (1) select tools based on patients’ cognitive-linguistic profile; (2) incorporate proxy reports from caregivers when appropriate; (3) utilize observational measures alongside self-report instruments; (4) implement multimodal assessment strategies combining subjective reports with objective performance measures; and (5) conduct serial assessments to account for symptom fluctuations and recovery progression.

## 5 Nursing intervention strategies for stroke patients with limb dysfunction

Effective rehabilitation interventions can reduce functional disability, improve patient satisfaction, and accelerate the recovery of stroke patients (81). However, only 15–20% of patients recover their pre-morbid function, and most patients experience persistent disability and require ongoing medical care and social support. Traditional treatments, including functional limb training, manual therapy, acupuncture, and electrical stimulation, have been widely used in clinical practice. In recent years, with the advancement of technology, rehabilitation techniques have been developed to address the physical impairments and psychological sequelae of patients with physical dysfunction.

### 5.1 Virtual reality in stroke rehabilitation

Virtual Reality (VR) has emerged as an emerging rehabilitation modality that offers healthcare providers innovative treatments and enhances patient engagement in therapy through immersive, multisensory environments that simulate real-world scenarios. Compared to traditional rehabilitation modalities, virtual reality technology offers rich environmental interactions, provides real-time performance feedback, and incorporates gamified tasks to motivate skill acquisition in patients with stroke. The effectiveness of VR aligns with Csikszentmihalyi’s Flow Theory, which posits that optimal engagement occurs when challenge levels match skill levels, resulting in a state of immersion and intrinsic motivation. VR environments facilitate this flow state by dynamically adjusting difficulty parameters to maintain patient engagement while progressively challenging motor capabilities. A study by In et al. demonstrated significant improvements in several metrics in the VR intervention group: the Berg Balance Scale (BBS), the Functional Reach Test (FRT), the Timed Upward Gaining and Walking (TUG) test (TUG), Postural Sway and 10-Meter Walk Test (10MWT) (82). However, the implementation of VR requires advanced hardware/software infrastructure, which limits clinical scalability due to high costs. Future large-scale, rigorously designed clinical trials are needed to validate its efficacy.

In a 3-week randomized controlled trial, Fan et al. divided 20 stroke patients into four groups: (i) virtual reality-based dual-task training (DVR), (ii) conventional treatment, (iii) placebo tabletop game, and (iv) no intervention. The results showed that the VR group had higher motivation for rehabilitation and better motor recovery. Similarly, Bergmann et al. conducted a 4-week trial (3 times per week) in which 20 subacute stroke patients were randomized to receive VR-enhanced robot-assisted gait training (RAGT) and standard RAGT (83). The VR-enhanced group demonstrated greater compliance, enhanced motivation for rehabilitation, and higher participation rates, underscoring the potential of VR to optimize therapy engagement.

### 5.2 Game-based therapy

Game-based therapy incorporates recreational elements into rehabilitation programs, leveraging reward-based learning models where intermittent reinforcement schedules optimize motivation and treatment adherence. This approach activates dopaminergic reward pathways, enhancing neuroplasticity and motor learning through increased repetition and engagement. The theoretical foundation is based on operant conditioning principles, where game achievements and progression serve as positive reinforcers for therapeutic movements. Lee et al. implemented a 4-week interactive video game-based gait training intervention in 12 patients with chronic stroke from two rehabilitation centers (53). Results showed that a gamified rehabilitation program improved exercise adherence and provided personalized motivational incentives in stroke survivors. Johar et al. implemented a 12-week game-based exercise therapy (including resistance training, dynamic balance training, and aerobic training) in 41 stroke patients. The test group utilized customized board games to achieve therapeutic goals, enhance multisensory stimulation and limb integration training, resulting in increased neuroplasticity and strengthened motivation for rehabilitation (84). Playing therapy in a rich participatory environment effectively mitigated cognitive decline, reduced anxiety and depression, and increased patient engagement in therapeutic activities.

### 5.3 Motivational interviewing

Motivational interviewing is a patient-centered, nonadversarial communication method designed to in quickly identifying and solving problems, autonomously regulating negative emotions, and independently modifying their interpersonal communication strategies. This approach is most effective in patients with mild to moderate cognitive impairment who retain sufficient executive function and communication abilities to engage in reflective dialogue. Patients with severe cognitive impairments, global aphasia, or anosognosia may benefit less from this verbally-mediated intervention. Chen et al. conducted a study in Taiwan, enrolling 72 first-time stroke patients, to evaluate the effects of motivational interviewing (85). Patients in the experimental group received motivational interviews once a week for 6 weeks, each lasting 15–30 min. In contrast, the control group received only routine care provided by a nurse, including the provision of stroke-related information, daily nursing support, and general checkups, for 3 weeks. The results showed that the experimental group was significantly more motivated to recover than the control group. Nurses implementing motivational interviewing require specialized training in reflective listening, open-ended questioning, and facilitating change talk to deploy this technique effectively within rehabilitation settings.

### 5.4 Film-based therapy

Film therapy utilizes film narratives, characters, and scenes to guide patients in exploring their emotional resonance with the film’s content, thereby alleviating psychological distress. This approach leverages observational learning principles through role modeling and vicarious reinforcement, where patients identify with characters overcoming similar challenges. While innovative, film therapy has implementation constraints, including literacy requirements, intact visual processing, and sufficient attention span, potentially limiting applicability in patients with severe cognitive deficits, visual impairments, or attention disorders. Kwon et al. conducted a randomized controlled trial that included 60 inpatient stroke patients undergoing rehabilitation (86). While the control group received standard care, the experimental group received a structured film intervention consisting of (i) disease-specific health education, (ii) viewing of 10 films demonstrating resilience and role modeling, and (iii) weekly 60 min group discussions for 10 weeks. The results showed that participation in therapeutic movies and group interactions facilitated subconscious self-exploration and enhanced motivation for adapting to and recovering from illness. For broader scalability, nursing protocols should include standardized film selection criteria, culturally appropriate content options, and simplified discussion guides adaptable to various cognitive levels. Integration with existing rehabilitation programs requires minimal additional resources, making this approach particularly suitable for resource-constrained settings.

### 5.5 Wearable devices

Wearable technology has become a frontier in rehabilitation medicine. Internet of Things (IoT)-based wearable health monitoring systems utilize sensor units to collect data on movement, electromyography (EMG), electrocardiography (ECG), and respiratory activity (87, 88). This data is transferred to a microprocessor and wirelessly transmitted to alarms, and feedback. Comprehensive physiological recordings of the cardiovascular, respiratory, and musculoskeletal systems enable clinicians to extract data sets and receive training feedback, thereby facilitating safer and more precise individualized rehabilitation programs. For effective nursing integration, clinical implementation requires: (1) structured training programs for nurses on device operation, data interpretation, and troubleshooting; (2) standardized protocols for incorporating wearable-generated data into clinical decision-making; (3) patient education materials explaining device functionality and self-monitoring procedures; and (4) interdisciplinary collaboration frameworks between nursing staff, physical therapists, and physicians. In a study by Si et al., wrist wearables were used to quantify goal-directed motor activity in daily life for post-stroke patients (89). Current limitations include sensor homogeneity, poor data accuracy, lack of functional versatility, and ergonomic challenges. Future advances require hardware-software optimization and an integrated stroke-specific wearable assessment and training ecosystem to provide comprehensive, intelligent, and efficient rehabilitation solutions.

Exoskeleton-assisted gait training devices utilize simulated physiological gait patterns to enhance walking in patients with physical dysfunction. These passive exoskeletons are operated parallel to the outside of the lower extremity via pulleys on the hip, knee, and ankle joints. During fitting, the exoskeleton is secured to the more affected limb using a ratcheting mechanism on a disc located above the waist, allowing for adjustable assistance until the user feels sufficient support to get off the ground. Clinical outcomes included improved gait symmetry, increased walking speed, enhanced stability and improved user confidence, with optimal efficacy observed up to 3 months post-stroke. Ergonomic design features ensure comfort and ease of donning and doffing, further promoting adherence (90). Moreover, nursing responsibilities include proper fitting procedures, monitoring skin integrity at contact points, and adjusting assistance levels progressively based on patient progress, which requires specialized competencies beyond traditional nursing education.

## 6 Conclusion

Rehabilitation motivation represents a critical determinant of functional recovery in stroke patients with limb dysfunction. This review synthesizes three key insights with significant clinical implications. First, the bidirectional relationship between psychological factors and rehabilitation outcomes necessitates integrated assessment approaches that capture both domains simultaneously. Specifically, kinesiophobia and self-efficacy emerge as modifiable psychological targets with substantial impact on functional recovery trajectories.

Second, motivation assessment tools demonstrate variable utility across different phases of stroke recovery. While clinician-rated instruments, such as the PRPS, offer practical efficiency in acute settings, comprehensive self-report measures, like the MORE scale, provide deeper insights during subacute and chronic phases when cognitive function stabilizes. The integration of both approaches yields the most comprehensive motivational profile to guide intervention planning.

Third, technological interventions (VR-based rehabilitation, wearable monitoring systems) show particular promise in addressing motivational barriers through their capacity to provide immediate feedback, progressive challenge calibration, and quantifiable achievement metrics. These approaches leverage neuroplasticity mechanisms while simultaneously addressing psychological engagement, representing a significant advancement beyond traditional rehabilitation paradigms.

Future research should prioritize developing personalized motivation enhancement protocols that integrate psychological interventions with technological innovations, tailored to patients’ specific motivational profiles, cognitive status, and recovery phase. This integrated approach holds the most significant potential for optimizing rehabilitation outcomes and improving quality of life for stroke survivors with limb dysfunction.
